# Editorial: Conflict and Cooperation in Microbial Societies

**DOI:** 10.3389/fmicb.2017.00141

**Published:** 2017-02-03

**Authors:** Ana E. Escalante, Michael Travisano

**Affiliations:** ^1^Laboratorio Nacional de Ciencias de la Sostenibilidad, Instituto de Ecología, Universidad Nacional Autónoma de MéxicoMexico City, Mexico; ^2^Department of Ecology, Evolution and Behavior, University of MinnesotaSt. Paul, MN, USA; ^3^Gortner Lab, BioTechnology Institute, University of MinnesotaSt. Paul, MN, USA

**Keywords:** conflict, cooperation, spatial structure, diversity, cheating, antagonistic interactions, mutualistic interactions, consortia

The most evident aspect of biodiversity is the variety of complex forms and behaviors among organisms, both living and extinct. Comparative molecular and physiological studies show that the evolution of complex phenotypic traits involves multiple levels of biological organization (i.e., genes, chromosomes, organelles, cells, individual organisms, species, etc.). Regardless of the specific molecular mechanisms and details, the evolution of different complex biological organizations share a commonality: cooperation (e.g., mutualisms) and conflict (e.g., antagonistic behavior) among the parts of the biological unit under study, so how do complex systems persist, given the necessity of cooperative behavior for their maintenance, when the potential for conflict occurs across all levels of biological organization?

In this Research Topic we are particularly interested in presenting ideas and work on the manifestation of different complex phenotypes that are based on cooperative or conflicting interactions of the component parts. Across these different systems, we see examples of cooperation and conflict at different levels of biological organization and discuss the consequences that this “tension” has had in the diversification and emergence of novel or complex phenotypic traits.

Cooperation among genes and among individual multicellular organisms (e.g., communities) are the extremes of different levels of biological organization in which cooperation has important roles in the origin and maintenance of complex phenotypic traits that range from the organization and size of genomes (Martínez-Cano et al.) to the maintenance of the microbiomes of animals (e.g., Loudon et al.). These models provide experimental data about the origin and maintenance of cooperative phenotypes and the role of cooperation in the evolution of biological complexity. In this Research Topic we present contributions that include all this range of biological complexity and that explore the mechanisms controlling the emergence and maintenance of diversity via cooperation in the face of conflict. We organized the contributions of this Research Topic in order of the level of biological organization that the studies refer to. We start with genome evolution (Martínez-Cano et al.), then continue with different examples of interactions among microorganisms from virus (Williams et al.) to bacteria, experimental and theoretical, and we finalize with an example of a multiple interaction system including animals, bacteria and fungi (Loudon et al.).

Martínez-Cano et al. present an analysis of experimental data on the processes behind genome reduction in endosymbiotic prokaryotes. Their work indicates that natural selection on cooperation or complementation of genomes (of the host and endosymbiont) may be behind this reduction which challenges other studies, based on comparative genomics, that have attributed this reduction to neutral processes (drift).

Mora van Cauwelaert et al. present a complementary view on the processes behind the emergence of multicellularity. Although the authors recognize the role of cooperative behavior, they discuss explicitly the role of other mechanisms such as physicochemical aspects of the environment in the emergence of complex phenotypes, such as multicellular aggregates in the early stages of the evolution of multicellularity.

A group of articles (Allison et al.; Kovács; Escalante et al.) discuss the role of spatial structure in the maintenance of cooperative behavior. Allison et al. focus their contribution in an experimental model of *Pseudomonas fluorescens* and the extracellular production of enzymes, a cooperative behavior that can only persist if spatial structure is imposed to the populations, in this way controlling the spread of cheater genotypes. Escalante et al. review studies on microbial population biology and identify that depending on the type of cooperative behavior (transitive or non-transitive), spatial structure may or may not favor the persistence of the cooperative behavior and the ecological diversity of the systems. Finally, Kovács, in a perspective article presents similar reflections on the role of spatial structure in the maintenance of mutualistic interactions and in turn of diversity.

Ponce-Soto et al. investigate experimentally the role of nutrient availability in the interactions and resulting diversity in microbial populations in natural environments. Their results suggest an important participation of environmental resource availability in shifting ecological strategies from more antagonistic (i.e., antibiotic resistance) when resources are limited to less so, maybe cooperative (i.e., biofilm formation), when the environment is enriched with nutrients. Moreover, in a complementary article, Aguirre-von-Wobeser et al. investigate theoretically the cost of production of antagonistic molecules in nutrient limited environments (oligotrophic) as a mechanistic exploration of interaction shifts in natural environments. Finally, Zapién-Campos et al. investigate through a mathematical model the dynamics of antagonistic interactions based on previously published experimental data, their results show that spatial structure emerge among the less antagonistic strains (more cooperative) as aggregates, which may be in accordance with the relevance of spatial structure in the emergence and maintenance of complex phenotypes and ecological diversity (see Allison et al.; Kovács; Escalante et al.).

Chubiz et al. study the mechanistic basis of cooperative and conflicting interactions at the genome-metabolite level by *in silico* knocking-out genes and testing the resulting metabolic interactions. Their findings highlight the importance of connecting genomic-metabolic studies to the context of interactions when looking for ecological stability or the mechanistic basis of the persistence of ecological diversity.

The last contribution of the research topic is presented by Loudon et al. showing the emergence of phenotypic traits at the microbial community level through the cooperative behavior of two bacterial populations in the production of antifungal metabolites in frogs' skin.

The maintenance of diversity and cooperation in the face of conflict has puzzled evolutionary biology for many years and in this topic we present new data and ideas that add to the mechanistic knowledge of the underlying processes. Mechanistic knowledge of any system or biological phenomenon allows for informed and effective manipulation or intervention of such. Thus, understanding the mechanisms of the origin and evolutionary maintenance of cooperation has implications far beyond evolutionary biology. Our research topic articles have the potential of impacting, for example, approaches in controlling microbial infections in medicine, as well as modes by which studies in synthetic biology are conducted when designing economically important microbial consortia.

## Author contributions

AE and MT contributed equally to the proposal and, organization of the Research Topic, as well as to the writing of the Editorial.

### Conflict of interest statement

The authors declare that the research was conducted in the absence of any commercial or financial relationships that could be construed as a potential conflict of interest.

